# Analysis of Prescriptions Dispensed After Death and Associated Medicare Spending Following Reimbursement Policy Changes in Medicare

**DOI:** 10.1001/jamanetworkopen.2023.14357

**Published:** 2023-05-25

**Authors:** Scott M. Vouri, Earl J. Morris, Yongkang Zhang

**Affiliations:** 1 Department of Pharmaceutical Outcomes and Policy, University of Florida College of Pharmacy, Gainesville; 2 Center for Drug Evaluation and Safety, University of Florida, Gainesville; 3 Department of Population Health Sciences, Weill Cornell Medical College, New York, New York

## Abstract

This cohort study examined the association of a prescription drug event edit policy change with prescriptions dispensed after the deaths of beneficiaries and estimated Medicare spending.

## Introduction

Reimbursement of Part D–covered medications dispensed to decedents may indicate wasteful health care spending.^1^ In July 2017, a prescription drug event (PDE) edit was enacted that reduced reimbursement of Part D–covered medications from 32 to 14 days after the deaths of community-dwelling Medicare beneficiaries whose prescriptions were dispensed by community or retail pharmacy services.^2^ Reimbursements remained up to 32 days after the deaths of those who were not community dwelling or who had prescriptions dispensed in other settings (eg, mail order pharmacy).

Current evidence of this wasteful spending is limited to HIV medications, and an evaluation of this PDE edit has not been conducted. We examined the association of this PDE edit policy change with prescriptions dispensed after the deaths of beneficiaries and estimated Medicare spending.

## Methods

In this cohort study, we used a 15% sample of 2016-2018 claims data for Medicare fee-for-service (FFS) beneficiaries aged 66 years or older. We included beneficiaries who died during the study period (decedents) (1) with 90 days or more of continuous enrollment of Medicare Parts A, B, and D before death; (2) who had a chronic condition^3^; and (3) who were classified as community dwelling based on the last dispensed prescription before death.

Outcomes included (1) number of decedents dispensed 1 or more prescriptions after death, (2) number of prescriptions dispensed after death, and (3) estimated Medicare spending (standardized to 2018 US dollars) per 1000 deaths. We used descriptive statistics to evaluate characteristics of decedents by whether they received 1 or more medications after death and by pharmacy settings among decedents who were dispensed prescriptions.

We conducted an interrupted time series (ITS) analysis (eMethods in Supplement 1). Among decedents, we compared prescriptions dispensed by a community or retail pharmacy, which were affected by the PDE edit, with prescriptions dispensed by other pharmacy settings,^4^ which were not affected by the PDE edit.^2^ Secondary post hoc analyses were conducted by stratifying decedents with or without chronic conditions (eMethods in Supplement 1). This study followed the STROBE reporting guideline,^5^ and the study protocol was reviewed and approved by the University of Florida institutional review board. Informed consent was not required because this was secondary use of administrative data.

## Results

A total of 331 632 decedents with a mean (SD) age of 78.0 (12.0) years were included, and 175 464 (52.9%) were female (Table). Overall, 4886 decedents were dispensed 1 or more prescriptions after death, with a decreased trend (15.7 vs 13.8 [before or after edit] beneficiaries per 1000 deaths). A total of 10 471 prescriptions were dispensed overall (35.8 vs 27.9 [before or after edit] prescriptions per 1000 deaths), leading to an estimated Medicare spending of $2 246 625 ($7847 vs $5824 [before or after edit] per 1000 deaths).

**Table.** Characteristics of Included Decedents Beneficiaries

Characteristic	Decedents, No. (%)				*P* value^b^
	Included (n = 331 632)	Dispensed ≥1 medication prescriptions (n = 4886)	Dispensed in community or retail pharmacy setting (n = 3352)^a^	Dispensed in other pharmacy settings (n = 1534)^a^	
Age, mean (SD), y	78.0 (12.0)	76.0 (13.1)	74.6 (14.0)	78.9 (10.3)	<.001
Sex					
Female	175 464 (52.9)	2633 (53.9)	1887 (56.3)	746 (48.6)	<.001
Male	156 168 (47.1)	2253 (46.1)	1465 (43.7)	788 (51.4)	
Race and ethnicity^c^					
White	277 763 (83.8)	3481 (71.2)	2144 (64.0)	1337 (87.2)	<.001
Black	31 849 (9.6)	750 (15.4)	616 (18.4)	134 (8.7)	
Other^d^	22 031 (6.6)	655 (13.4)	592 (17.7)	63 (4.1)	
Region					
Northeast	47 955 (14.5)	790 (16.8)	542 (16.2)	248 (16.2)	<.001
Midwest	54 172 (16.3)	693 (14.2)	384 (11.5)	309 (20.1)	
South	188 191 (56.8)	2738 (56.0)	1979 (59.0)	759 (49.5)	
West	41 325 (12.5)	665 (13.6)	447 (13.3)	218 (14.2)	
Alzheimer disease	121 321 (36.6)	1957 (40.0)	1456 (43.4)	501 (32.7)	<.001
Cardiovascular disease	216 904 (65.4)	3404 (69.7)	2324 (69.3)	1080 (70.4)	.45
Chronic lung disease	128 390 (38.7)	2101 (43.0)	1510 (45.0)	591 (38.5)	<.001
Atrial fibrillation	80 138 (24.2)	1105 (22.6)	652 (19.5)	453 (29.5)	<.001
Solid cancer	65 332 (19.7)	739 (15.1)	466 (13.9)	273 (17.8)	.004
Heart failure	176 770 (53.3)	2935 (58.0)	1931 (57.6)	904 (58.9)	.38
Chronic kidney disease	215 908 (65.1)	3400 (69.6)	2374 (70.8)	1026 (66.9)	.006
Depression	96 603 (29.1)	1745 (35.7)	1281 (26.2)	464 (30.3)	<.001
Diabetes	147 231 (44.4)	2695 (55.2)	1929 (57.6)	766 (49.9)	<.001
Hyperlipidemia	182 116 (54.9)	2882 (59.0)	1879 (56.1)	1003 (65.4)	<.001
Hypertension	262 576 (79.2)	4101 (83.9)	2791 (83.3)	1310 (85.4)	.06
Hypothyroid	75 575 (22.8)	1155 (23.6)	698 (20.8)	457 (29.8)	<.001
Osteoporosis	29 410 (8.9)	420 (8.6)	277 (8.3)	143 (9.3)	.22
Arthritis	145 683 (43.9)	2419 (49.5)	1731 (51.6)	688 (44.9)	<.001

^a^ Not mutually exclusive as patients may have drugs dispensed from both pharmacy settings.

^b^ Comparison of deceased beneficiaries dispensed in community or retail pharmacy setting vs other pharmacy settings.

^c^ We used the race code from the Medicare Master Beneficiary Summary File.

^d^ An aggregate of Asian, Hispanic, North American Native, other, and unknown.

Nonsignificant reductions in beneficiaries receiving 1 or more prescriptions per 1000 deaths (change in level, −0.440 [95% CI, −2.395 to 1.516]; *P* = .66), prescriptions dispensed per 1000 deaths (change in level, −4.956 [95% CI, −11.541 to 1.630]; *P* = .14), and estimated Medicare spending per 1000 deaths (change in level, −$1375.16 [95% CI, −$5435.30 to $2684.99]; *P* = .50) associated with the Part D PDE edit (Figure) were identified from the ITS analysis. In secondary analyses, significant reductions in prescriptions dispensed per 1000 deaths (change in level, −8.000 *P* = .049) and estimated Medicare spending per 1000 deaths (change in level, −$3309.74; *P* = .04) associated with the Part D PDE edit for patients with hyperlipidemia were identifed; however, there was overlap in 95% CIs in these 2 measures for beneficiaries without hyperlipidemia. There were no differences between patients with and patients without Alzheimer disease and atrial fibrillation.

**Figure.**
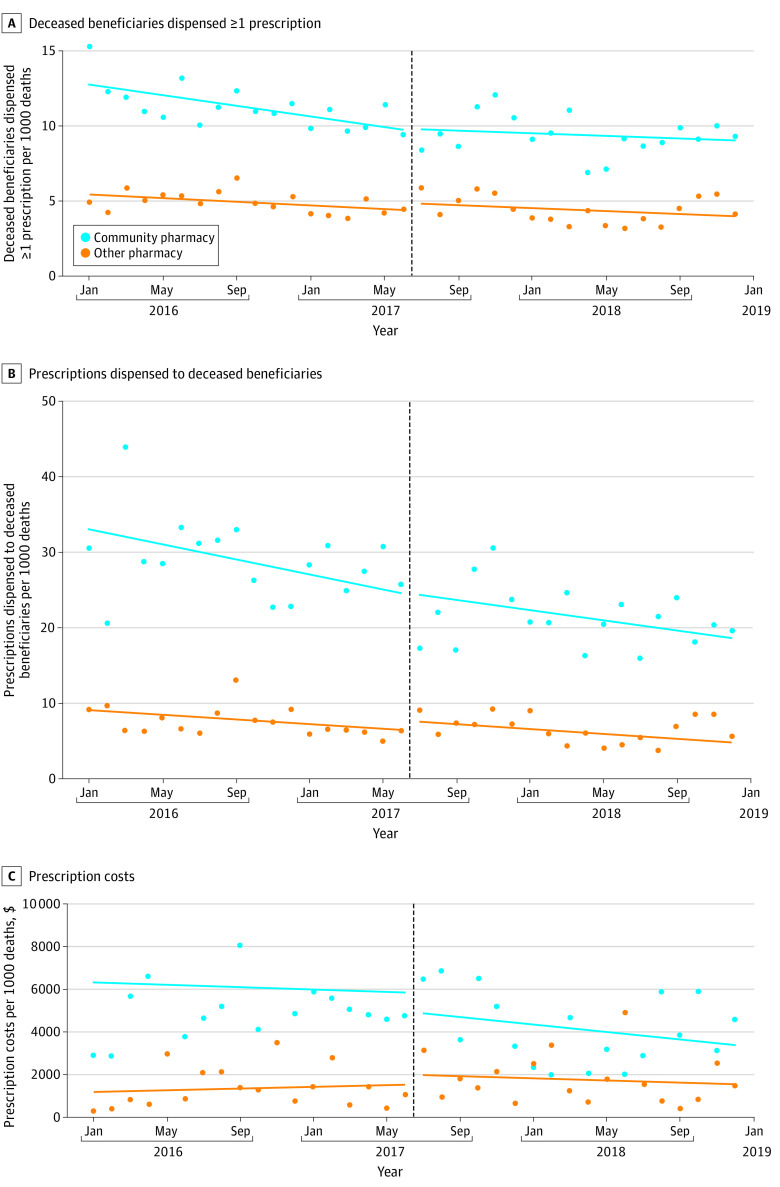
Association Between Prescription Drug Event Edit and Prescription Dispensing to Deceased Medicare Beneficiaries A, There was a nonsignificant reduction of 0.44 deceased Medicare beneficiaries per 1000 deaths (−0.440 [95% CI, −2.395 to 1.516]) who were dispensed 1 or more prescriptions immediately following the prescription drug event edit that went into effect on July 1, 2017 (as signified by the vertical dashed line). B, There was a nonsignificant reduction of 5 prescriptions dispensed per 1000 Medicare beneficiary deaths (−4.956 [95% CI, −11.541 to 1.630]) immediately following the prescription drug event edit that went into effect on July 1, 2017 (as signified by the vertical dashed line). C, There was a nonsignificant reduction of $1375 in Medicare spending per 1000 Medicare beneficiary deaths (−$1375.16 [95% CI, −$5435.30 to $2684.99]) immediately following the prescription drug event edit that went into effect on July 1, 2017 (as signified by the vertical dashed line).

## Discussion

Following the PDE edit, 13.8 beneficiaries per 1000 deaths were dispensed a prescription after death, resulting in 27.9 prescriptions and $5824 in estimated Medicare spending per 1000 deaths. Extrapolations using 2019 Medicare death data^6^ indicated that up to 29 000 beneficiaries would be affected, up to 60 000 prescriptions would be dispensed after death, and up to $12.4 million in spending. Although nonsignificant in our model, it is estimated that this edit was associated with an estimated reduction in spending of $2.9 million.

Limitations of this study included data that were limited to deceased community-dwelling Medicare FFS beneficiaries. Findings may differ for Medicare Advantage beneficiaries or those who are not community dwelling, and estimated Medicare spending does not include rebates, risk sharing, or gap discounts. This cohort study found nonsignificant, although potentially meaningful, reductions in the number of deceased beneficiaries who were dispensed a prescription, in the total number of prescriptions dispensed, and in Medicare spending associated with a Part D PDE edit.

## Notes

Supplement 1.
eMethods.

Supplement 2.
Data Sharing Statement
