# Supplementary material for: Analysis of Prescriptions Dispensed After Death and Associated Medicare Spending Following Reimbursement Policy Changes in Medicare
Source: JAMA Netw Open. 2023 May 25;6(5):e2314357. doi: 10.1001/jamanetworkopen.2023.14357 (PMC10214031; doi:10.1001/jamanetworkopen.2023.14357)
Supplement: Supplement 1. — eMethods. [file jamanetwopen-e2314357-s001.pdf]

# Supplemental Online Content

Vouri SM, Morris EJ, Zhang Y. Analysis of prescriptions dispensed after death and associated Medicare spending following reimbursement policy changes in Medicare. *JAMA Netw Open*. 2023;6(5):e2314357. doi:10.1001/jamanetworkopen.2023.14357

**eMethods.**

This supplemental material has been provided by the authors to give readers additional information about their work.

© 2023 Vouri SM et al. *JAMA Network Open.*

**eMethods.**

The interrupted time series method fitted generalized least squares linear models. This method of analysis evaluates level (immediate change) and trend (change over time) change before and after the prescription drug event (PDE) edit was enacted in a group impacted by the policy (i.e., decedents whose medications were dispensed from a community/retail pharmacy) relative to level and trend change before and after the PDE edit was enacted in a group not impacted by the policy (i.e., decedents whose medications were dispensed from a non-community/retail pharmacy setting). Autoregressive–moving average correlation structure using Durbin-Watson tests were performed over 12 lag periods. No autocorrelation was noted in any of the three analyses. A 2-sided $P < .05$ was considered statistically significant. The study was approved by the University of Florida institutional review board and followed the STROBE reporting guideline. All analyses were completed between December 2021 and July 2022.

In secondary post hoc analyses, we conducted stratified analyses on decedents with and without three chronic conditions with the largest absolute differences between those dispensed in Community/Retail Pharmacy Settings and Other Pharmacy Settings (Alzheimer's disease (10.7%), Atrial Fibrillation (10.0%), Hyperlipidemia (9.3%)) to evaluate the association between these chronic conditions and each of the outcomes. Differences were determine based on non-overlapping confidence intervals.

© 2023 Vouri SM et al. *JAMA Network Open.*
