# Supplementary material for: Analysis of Prescriptions Dispensed After Death and Associated Medicare Spending Following Reimbursement Policy Changes in Medicare
Source: JAMA Netw Open. 2023 May 25;6(5):e2314357. doi: 10.1001/jamanetworkopen.2023.14357 (PMC10214031; doi:10.1001/jamanetworkopen.2023.14357)
Supplement: Supplement 2. — Data Sharing Statement [file jamanetwopen-e2314357-s002.pdf]

Data Sharing Statement

Vouri. Analysis of Prescriptions Dispensed After Death and Associated Medicare Spending Following Reimbursement Policy Changes in Medicare. *JAMA Netw Open*. Published May 25, 2023. doi:10.1001/jamanetworkopen.2023.14357

Data
Data available: No

Additional Information
Explanation for why data not available: Limited by the DUA with CMS
